# Pod Dehiscence in Soybean: Genome Wide Association Study and Genomic Prediction

**DOI:** 10.3390/plants14223505

**Published:** 2025-11-17

**Authors:** Shynar Mazkirat, Kulpash Bulatova, Svetlana Didorenko, Sholpan Bastaubayeva, Dilyara Babissekova, Sholpan Khalbayeva, Azamat Tukenov, Akzhan Yespembetova, Nurgul Saparbayeva, Yuri Shavrukov

**Affiliations:** 1 Kazakh Research Institute of Agriculture and Plant Growing, Almalybak 040909, Almaty District, Kazakhstan; shynarbek.mazkirat@gmail.com (S.M.); svetl_did@mail.ru (S.D.); sh.bastaubayeva@kazapg.kz (S.B.) dilyara280188@mail.ru (D.B.); sholpan_2706@mail.ru (S.K.); tukenov97@mail.ru (A.T.); akzhanes@mail.ru (A.Y.); n.saparbaeva@mail.ru (N.S.); 2 College of Science and Engineering, Biological Sciences, Flinders University, Adelaide, SA 5042, Australia

**Keywords:** DArT markers, genome-wide association study (GWAS), genomic prediction, pod dehiscence, soybean

## Abstract

Pod dehiscence is one of the main factors which play a vital role on the final yield of many crops including soybean and, therefore, it is important to elucidate genetic mechanisms associated with this trait. In this study, morphological, physiological and biochemical analysis was conducted for pod and pod-related traits on 170 soybean genotypes with diverse origins. Subsequently, a genome-wide association study (GWAS) was performed using Silico-DArT and DArT SNPs markers. In total, 48 QTLs were identified with 14 stable QTLs, mostly located on chromosomes 6, 13 and 16, corresponding to pod dehiscence and pod-related traits. From putative candidate genes, two most stable and important genes for pod dehiscence with known functions were emphasised from the QTLs: Glyma.13G184500 and Glyma.16G141100, encoding transcription factors DNA-binding bromodomain-containing protein and C2H2 zinc finger protein, respectively. Finally, a genomic prediction approach was implemented to select genotypes most resistant to pod dehiscence. GWAS-derived markers confirmed the stable prediction of pod dehiscence in studied accessions from different populations and the best non-dehiscent soybean genotypes were successfully selected.

## 1. Introduction

A major source of high-quality protein and oil for human diets and animal feed, soybean [*Glycine max* (L.) Merr.] plays a crucial role in ensuring global food and nutritional security. The demand for soybeans has increased due to the world’s expanding population, underscoring the necessity of developing high-yielding, stress tolerant and nutritionally improved cultivars [1]. Soybean can grow in a variety of climatic and agronomic conditions and its total cultivated area is about 122 million hectares, producing over 353 million metric tons annually. It is mainly grown in Brazil, the USA, Argentina, China and India [2,3,4].

In Kazakhstan, the area under soybean cultivation is increasing progressively due to rising commercial interest. For example, since 2000 in Kazakhstan, the soybean total growing area increased from 3500 ha to nearly 128000 ha in 2022 [5]. The main soybean sowing area in Kazakhstan includes the Almaty and Zhetysu regions [6]. However, the harvesting period in these regions overlaps with strong drought conditions, increasing seed loss by pod dehiscence.

Pod dehiscence (PD), or pod shattering, in wild soybean or landraces is an important trait for plant populations in natural environments [7,8]. In domesticated crops, PD is an entirely unfavourable trait due to the detrimental impact it has on seed yield during harvest [9]. Therefore, the production of genotypes with no or minimal PD is strongly emphasised in all soybean breeding programs. Resistance to PD is a complex of physical, physiological and biochemical processes [10], and is associated with the pod-related traits: pod length, width and thickness as well as ratios, including length/width, length/thickness and width/thickness. Usually, PD occurs when pods reach the stage of physiological maturity. One of the reasons relates to decreased humidity in pod walls and reduced relative water content in the pod [11,12,13,14,15]. However, there are few investigated cases of pod opening, which occurs when the pods are still immature and green. Water deficit can be a trigger to immature pod opening in soybean, as reported in experiments in greenhouse conditions studying the effect of drought on plants during pod formation [16].

The chlorophyll content is an important trait to estimate crop growth, and photosynthetic rate [17,18]. Using a Minolta 502 SPAD meter (Konica Minolta, Tokyo, Japan) for rapid measurement of chlorophyll content in soybean plant leaves, it was found that the chlorophyll content remained stable from stage R2 (full flower) till stage R5 (beginning of seed formation) but declined after that [19]. The content of mineral elements Mg and Fe in the pod valves of grain soybeans at the R5 and R6 stages negatively correlates with PD. These elements participate directly in photosynthesis and chlorophyll synthesis, increasing their level and pod resistance to dehiscence. Presumably, the high concentration of chlorophyll in soybean plants at the pod filling stage may be an indicator for resistance to PD [20].

Numerous yield parameters can be associated with PD. For example, a soybean genotype with small pods was shown to have less seeds and non-dehiscent pods [21]. Additionally, the percentage of PD was positively correlated with the number of productive branches, 100-seed weight and some pod-related traits [20]. Plant height was also shown to be associated with PD. For example, taller and stronger plants from the genus *Brassica* were more resistant to dehiscence and shattering of siliques [22,23]. Soybean cultivars resistant to PD showed a tendency to have a longer stem with more nodes, lower height to first pod and smaller seed size [24].

Heat stress causes excessive generation of reactive oxygen species (ROS), which harms intracellular machinery [25,26]. Antioxidant enzymes, including superoxide dismutase (SOD), catalase (CAT), peroxidase (POX), and ascorbate peroxidase (APX), play an important role in neutralizing excess ROS and reducing their detrimental effects [27,28]. It was confirmed that drought stress during the flowering and pod development stages had a severe effect on PD in *Brassica napus* L. [29], and a positive correlation between PD and antioxidant enzymes such as SOD and CAT were reported recently [30]. Additionally, the activity of antioxidant enzymes SOD, POD and CAT was shown to strongly correlate with each other [31]. Among these enzymes, SOD has the greatest importance for active oxygen scavenging [32,33]. Therefore, that could be sufficient to determine the changes in the activity of one of these enzymes, for example SOD, and similar trend in the activity of other antioxidant enzymes is expected. Therefore, increased activity of antioxidant enzymes in plants can help to support tolerance of plants to stress factors.

The study of Quantitative trait loci (QTL) is often used in plant biology to identify genes of interest [34,35]. For PD in soybean, the first QTL was reported in chromosome 16 using Restriction fragment length polymorphism (RFLP) markers [36]. This QTL was later identified and mapped in the genetic region between SSR markers Sat_093 and Sat_366, and it was named qPDH [37]. Several other unique QTLs were discovered on chromosomes 1, 5, 8 and 14 using the Specific-locus amplified fragment sequencing method (SLAF) [38]. The major QTL, *pdh1* (Glyma.16G141400) linked to PD was revealed in cultivated soybean [9]. In the same chromosome 16, the *SHAT1-5* gene, a NAC Transcription Factor (Glyma.16G019400) was identified as an activator of secondary wall biosynthesis, promoting thicker fiber cap cells in wild soybean accessions [39]. Bi-parental populations with various genetic origins were used in several studies making the linkage map of the *qPDH1* locus. Additionally, several minor QTLs were also found in chromosomes 2, 5, 8, 10, 14 and 19 [36,40]. An RNA-sequencing approach was successfully used for QTL identification in soybean chromosome 19 [41].

Genome wide association study (GWAS) represents another advanced method of QTL identification based on the comparison of genotyping and phenotyping results in diverse sets of germplasms in different environments rather than segregating hybrid populations [42,43]. In soybean, GWAS application resulted in the discovery of several more QTLs for PD dispersed throughout the genome [44]. In total, 163 SNPs were identified and most of them (136 SNPs) were linked to the known qPDH1 region. Other QTLs were found on chromosomes 1, 4, 6, 8, 9, 11, 16, 17, 18 and 20, and among them, Glyma09G06290 corresponding to a bHLH family transcription factor was suggested to be the most important putative candidate gene linked to PD. Most recently, GWAS has helped to identify 86 QTLs associated with PD on all chromosomes across four *G. max* populations. Among them, eight important QTLs linked to PD were revealed on chromosomes 1, 3, 4, 10, 11, 17 and 19. These eight genes were homologous to *Arabidopsis thaliana* genes which played important roles in PD through the lignification, breakdown, and biosynthesis of the cell wall [45]. Special attention was paid to soybean chromosome 16, where one main QTL was identified and titled qPS-DS16-1 with the candidate gene Glyma.16G076600, encoding Cytochrome P450, which contributed to PD resistance in soybean [46]. Recently, gene *Sh1* (Glyma.16G141100), the repressor of *SHAT1-5*, was found in the same chromosome 16. This *Sh1* gene encoded a C2H2-like zinc finger transcription factor and was found to cooperate with another gene *Pdh1*, which encodes a dirigent-like protein. The partner genes, *Sh1* and *Pdh1*, were reported to strongly regulate *SHAT1-5*, making them major controllers of PD in soybean [47].

As mentioned above, PD is correlated with many other traits and, therefore, controlled by multiple structural and regulatory genes, which play significant roles in the development of cell-walls in pods. Numerous minor loci along with major QTLs are also involved in the control of PD. Genomic prediction (GP) is a powerful method to reveal valuable information in complex traits rather than marker-assisted selection (MAS) [48,49]. GP utilizing high density markers across the genome can accelerate the breeding process and enable the integration of multiple environment data for predicting crop traits [50]. GP has been used widely to improve the genetic gain for various traits in many crops including soybean. For instance, GP was implemented for enhancing yield in soybean [51,52], disease resistance [53] and tolerance to abiotic stresses [54]. These findings emphasize the potential of GP in speeding up crop genetic improvement.

Despite some attempts of genetic analyses conducted to identify the genes controlling resistance to PD, the fundamental biological mechanisms of this trait remain unclear. Some studies used very limited genotypes, while others used low-density molecular maps for the analysis. Therefore, it is important to conduct Diversity Arrays Technology analysis (DArT) using GWAS with high-density SNP panels in sufficient and diverse soybean germplasm accessions.

The aims in this study were: (1) Analysis of genomic regions corresponding to PD based on GWAS with DArT markers in 170 soybean genotypes with diverse origins; (2) Identification of potential candidate genes linked to PD; (3) Prediction and selection of genotypes resistant to PD for soybean breeding programs using genomic prediction.

## 2. Materials and Methods

### 2.1. Plant Material and Field Trials

In the current study, 170 soybean accessions with different levels of PD and diverse origins were studied. The population size of 170 accessions is considered as moderate for plant research, but still acceptable for GWAS. This point can be supported by genetically very diverse accessions mentioned above, high-quality DArT markers that passed strict quality-control filters, described in Section 2.4 below, and rigorous logarithm of odds threshold used in this study and described in the following Section 2.6. The major germplasm collection of soybean cultivars originated from the Kazakh Research Institute of Agriculture and Plant Growing (KRIAPG), Almaty district, Kazakhstan. Other genotypes were received from germplasm collections of N.I.Vavilov Research Institute of Plant Industry, St.Petersburg, Russia; V.S.Pustovoit All-Russian Research Institute of Oilseeds, Krasnodar, Russia; Siberian Research Institute of Fodder Crops, Novosibirsk region, Russia; Krasnoyarsk Research Institute of Agriculture, Krasnoyarsk, Russia; Research Production Association ‘Soya-Center’, Minsk region, Belarus; Soybean Research Institute, Poltava region, Ukraine; US National Plant Germplasm System (USDA), Urbana, Illinois, USA; and the Research Institute of Field and Vegetable Crops, Novi Sad, Serbia. The full list of the accessions used is present in Supplementary Table S1.

Two sowing dates were used: the traditional date (late April or early May) and a delayed planting (late May or early June), to create different environmental conditions during the growing season. The field trials were conducted over two years (2023–2024) at the research fields of KRIAPG, located in South-Eastern Kazakhstan, at a latitude of 43° N. Seeds were sown to study their performance under four different environments: (1) early sowing in 2023; (2) late sowing in 2023; (3) early sowing in 2024; and (4) late sowing in 2024. The experiment was carried out using a randomized complete block design (RCBD) with three replications. The climate during the soybean sowing season of 2023 was characterized as hot with low rainfall, while 2024 was also hot but with heavy rainfall. The maximum summer air temperature reached nearly 34 °C in 2023 compared to 31 °C in 2024. In general, the climate in the region is continental with hot summers and humid winters, and estimated as ‘Dfa’, according to the Köppen classification [55]. The average temperature in late April is about 12–13 °C, while in early June it is 20–21 °C, and annual precipitation ranges from 100 to 60 mm (late April and early June, respectively).

### 2.2. Morphological, Physiological and Biochemical Characteristics of Pods in Soybean Accessions

Morphological traits related to PD such as pod length (PL), pod width (PW) and pod thickness (PT) were measured on 10 pods on the main stem in five plants using a calliper. The data received were used for calculation of ratios in pods: length/width ratio (LWR), length/thickness ratio (LTR) and width/thickness ratio (WTR).

Plant material (leaves and pods) for physiological and biochemical analyses was collected at the plant development stages R4–R5 and stored at −70 °C until used for assays. Chlorophyll and carotenoids were extracted from leaves using 80% acetone following the protocol published earlier [56]. The concentration of chlorophyll a and b, as well as carotenoids was measured at wavelengths of 646.8, 663.2 and 470 nm, respectively, using a Jenway 635031 spectrophotometer (Bibby Scientific, Staffordshire, UK).

The relative water content (RWC) was measured according to the protocol published earlier [57]. For this analysis, immature pods from at least four plants for each sample taken in the field were placed in a container with cooling elements and quickly delivered to the laboratory. After removing the seeds, pods were weighed immediately to determine fresh weight (FW) and placed in deionized distilled water for 4 h. Then, pods were weighed again to obtain turgid weight (TW), and they were then dried in an oven at 80 °C for 24 h. Fully dried pods were reweighed to determine dry weight (DW). RWC was calculated by the following formula:

$$RWC=\frac{\left(FW-DW\right)}{\left(TW-DW\right)}\times 100\%$$

Extraction of the antioxidant enzymes superoxide dismutase (SOD) and polyphenol oxidase (PPO) was performed with a phosphate buffer containing 0.25 mM ethylenediaminetraacetic acid (EDTA), polyvinylpyrrolidone 25 (PVP-25, 2%, *w*/*v*), 1 mM ascorbic acid and glycerol (10%, *w*/*v*), according to a previously published protocol [58]. SOD activity was determined by photoreduction inhibition of nitroblue tetrazolium (NBT) according to [59] and expressed in units/g FW. This was used as substrate to determine PPO activity, which was expressed similarly as units/g FW of pyrocatechol [60].

### 2.3. Assessment of Pod Dehiscence

The oven-dry method was used to assess the degree of PD. All mature pods were harvested from plants at the same maturing stage (R8) and stored at room temperature for one week. After that, the PD ratio in each accession with three biological replicates was calculated as the proportion of dehiscent pods to the total number of pods after 12 h of drying at 80 °C in the oven (Drying chamber ED-115, BINDER, Tuttlingen, Germany) The percentage of PD was determined according to a 1–5 scale, where 1 represents 0% dehiscent pods; 2, 1–10%; 3, 11–25%; 4, 26–50% and 5 represents 50% dehiscent pods [61]. Based on this scale, the PD phenotypes were classified as follows: 1, very resistant; 2, resistant; 3, moderately resistant; 4, moderately sensitive; and 5, very sensitive to dehiscence.

### 2.4. DArTseq Genotyping and Marker Quality Control

DNA was extracted from young leaf tissue of 170 soybean accessions using a DNeasy Plant Mini kit (Qiagen, Hilden, Germany). The extracted DNA was diluted to a concentration of 100 ng/μL, and 50 μL of high-quality DNA from each genotype was submitted to Diversity Arrays Technology (DArT Pty Ltd., Canberra, Australia) (https://www.diversityarrays.com; accessed on 20 October 2024) for whole-genome analysis using DArT markers. This technology is based on sequencing of an enhanced library using a Next generation sequencing (NGS) platform with genome complexity reduction, and 60K DArT clones were applied for the study. Results of genotyping with 18,540 Silico-DArT and 41,310 DArT-SNP markers were received. Based on genome assembly, markers with unknown chromosome positions were removed from the analysis. The remaining Silico-DArT and DArT-SNP markers used for the association analysis were filtered based on a call rate of ≤80%, marker reproducibility at ≤95%, minor allele frequency (MAF) ≤ 5%, and missing observation fractions ≥ 10%.

### 2.5. Population Structure Analysis

STRUCTURE v2.3.3 software was applied to assess population structures utilizing a Bayesian-Markov chain-Monte Carlo (MCMC) method grounded in admixture and correlated allele frequencies [62]. The data set was run through 10,000 Markov chain-Monte Carlo iterations with an initial burn-in period of 10,000 with five replicates, considering several subgroups (K) ranging from 1 to 10. The python script of Structure Harvester ‘StructureHarvester.py’ [63] was used to determine the optimal k-value [64], as well as to illustrate the results obtained from STRUCTURE v2.3.3.

### 2.6. GWAS Analysis, QTL and Candidate Gene Prediction

To identify SNPs with significant linkage to the studied yield-related soybean traits, GWAS was conducted using the Genome association—Prediction integrated tool (GAPIT), version 3, with several models with increased power and accuracy for genome association [65]. The GAPIT models used in this study include the Bayesian-information and Linkage-disequilibrium iteratively nested keyway (BLINK) [66], the Fixed and random model circulating probability uniform (FarmCPU) [67], the Multiple loci mixed model (MLMM) [68], the Mixed linear model (MLM) [69], and the General linear model (GLM) [70]. To identify highly significant associations, in this study, rigorous logarithmic of odds (LOD ≥ 6.0) criteria were used, based on the Bonferroni correction test (α = 0.05) [71]. 20 kb genomic flanking regions were used to detect a candidate for each molecular marker, and annotation was based on the soybean reference genome Williams 82 (Wm82.a2.v1). The chromosome fragments for ±20 kb and even less were often used in similar studies [72]. Protein database UniProt was also implemented to find protein functions of detected QTLs (https://www.uniprot.org; accessed on 10 July 2025).

### 2.7. Genomic Prediction (GP) for Genomic Selection of Pod Dehiscence

The fallowing six models were used to perform GP: Genomic best linear unbiased prediction (gBLUP) [73], Bayes LASSO (BL) [74], marker-assisted BLUP (maBLUP) [75], Random Forest (RF) [76], Reproducing Kernel Hilbert Space regression (RKHS) [77], and ridge regression best linear unbiased prediction (RR-BLUP) from the rrBLUP package [78]. GP was conducted for two marker sets. The first marker set included all markers from 170 soybean genotypes for predicting genomic estimated breeding values (GEBV) in GP, while the second marker set contained only 51 GWAS-derived markers. Two scenarios were used for GP using each marker set in the GAPIT package (version 3.5.0): (1) A five-fold cross-validation approach was implemented, 80% of all genotypes were used as the training set, and the remaining 20% of genotypes were employed as the testing set. (2) the population was divided into two subgroups (1 and 2) according to their origin. Subgroup1 included all genotypes except genotypes from North America and China, and this subgroup-1 was employed as the training set. Conversely, subgroup-2 containing all genotypes from both North America and China was used as the testing set. The accuracy of genomic selection was derived from the results of 100 replications and compared between GP models and illustrated by boxplots generated using the ggplot2 package in R version 4.5.1.

### 2.8. Statistical Analysis

All statistical analysis and data visualizations were performed in R version 4.5.1 [79]. Boxplots and histograms were illustrated using the ‘ggplot2 package’. Pearson correlation analysis was conducted using the R package ‘Performance Analytics’, and multiple testing correction was applied using the FDR method. The R function was used for one-way ANOVA.

## 3. Results

### 3.1. Pods and Pod-Related Agronomic Traits

The PD in 170 soybean accessions was evaluated along with associated pod-related traits (PL, PW, PT, LWR, LTR and WTR), as well as photosynthetic pigments, antioxidants, and RWCP parameters (Table 1). The PD levels in the most sensitive and most resistant genotypes were 88.25% and 0%, respectively. The average PL was approximately 4.53 cm, with the shortest measuring 3.37 cm and the longest reaching up-to 6.8 cm. PW ranged from 0.7 to 1.21 cm, while the PT varied between 0.52–0.84 cm.

**Figure 1:**
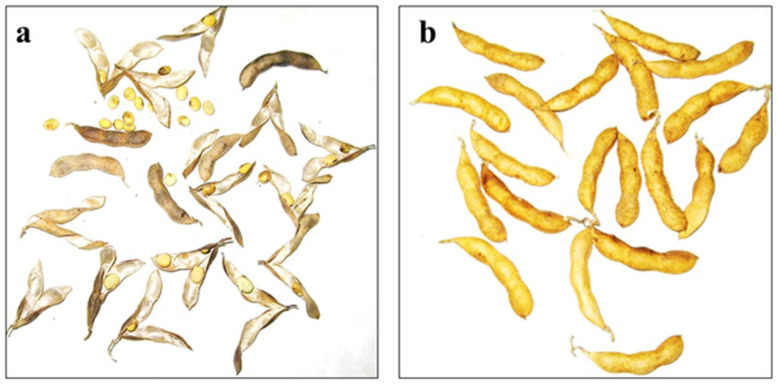
Pod dehiscence in soybean genotypes: (**a**) cv. Kollekcyina with very high level of pod dehiscence; (**b**) cv. Zispida with low level of pod dehiscence.

**Figure 2:**
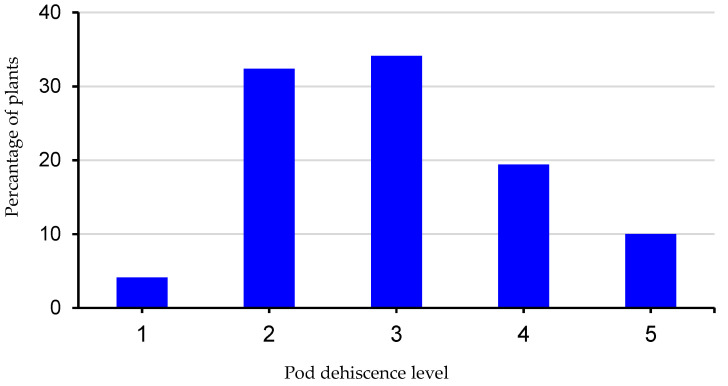
Distribution of 170 soybean accessions according to their pod dehiscence level: 1, very resistant; 2, resistant; 3, moderately resistant; 4, moderately sensitive; and 5, very sensitive.

After treatment at 80 °C, almost all pods were open in soybean genotypes sensitive to PD, whereas no opening pods were observed in accessions highly-resistant to PD (Figure 1). According to the results of the oven-dry methods, 170 soybean samples were classified into five groups (Figure 2), including 4.1% very resistant, 32.4% resistant, 34.1% moderately resistant, 19.4% moderately sensitive, and 10% very sensitive to PD.

**Figure 3:**
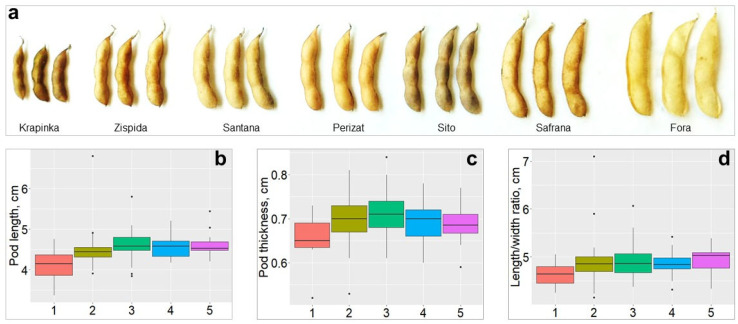
Pod-related traits and their distribution across various PD groups. (**a**) pod images from smallest to largest in size with corresponding PD: Krapinka, very resistant; Zispida, resistant; Santana and Perizat, moderately sensitive; Sito and Safrana, moderately resistant; Fora, very sensitive. Variability of PL (**b**): PT (**c**) and LWR (**d**) are shown in soybean accessions distributed among five PD groups: 1, very resistant; 2, resistant; 3, moderately resistant; 4, moderately sensitive; and 5, very sensitive.

The studied agronomic traits potentially involved in PD were compared across the five groups of genotypes. As a result, three traits, pod length, pod thickness, and length/wide ratio of pods, showed highly significant differences in soybean accessions with contrasting levels of PD (Figure 3). Genotypes belonging to high-resistance Group 1 exhibited significantly lower values of PD compared to those accessions in other Groups. For instance, the average pod length was 4.1 cm in Group 1, whereas in other Groups 2–5, it ranged between 4.57 and 4.61 cm (*F* = 4.021, *p* = 0.0038). The pod thickness in high-resistance Group 1, was 0.65 cm, while it was around 0.70 cm in other Groups on average (*F* = 3.261, *p* = 0.0132). The length/width ratio of pods in Group 1 was 0.463 units, whereas in highly-sensitive Group 5, it reached up to 0.495 units (*F* = 2.838, *p* = 0.0261).

**Figure 4:**
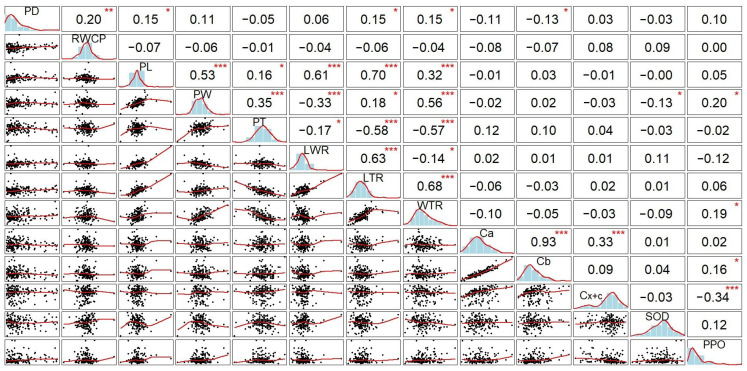
Pearson’s correlation coefficients for 13 studied pod-related traits in 170 soybean genotypes. Abbreviations: PD, pod dehiscence; RWCP, relative water content in pod; PL, pod length; PW, pod width; PT, pod thickness; LWR, length/width ratio; LTR, length/thickness ratio; WTR, width/thickness ratio; Ca, chlorophyll a; Cb, chlorophyll b; Cx+c, total carotenoids; SOD, superoxide dismutase, and PPO, polyphenol oxidase. In this correlation matrix, the lower triangle displays scatterplots, the upper triangle reports the correlation coefficients, and the diagonal shows the density distributions. Asterisks indicate statistical significance: * *p* ≤ 0.05, ** *p* ≤ 0.01, *** *p* ≤ 0.001.

The correlation analysis indicated that RWCP, PL, LTR and WTR pod-related traits showed significant positive correlations with pod dehiscence level (*r* = 0.20, 0.15, 0.15 and 0.15, respectively) (Figure 4). This may indicate that these pod-related traits have a strong effect on PD in soybean, and the lower values of these traits are related to cultivars with a high level of dehiscence. In contrast, negative association between PD and Cb was observed in the current study, suggesting that photosynthesis is more active in non-dehiscent genotypes. However, no significant correlations of PD with antioxidants were found. Nevertheless, antioxidants remain the most crucial components to prevent PD by regulating ROS levels that have a negative effect on the PD zone.

### 3.2. DArTseq Genotyping and Marker Quality Control

**Figure 5:**
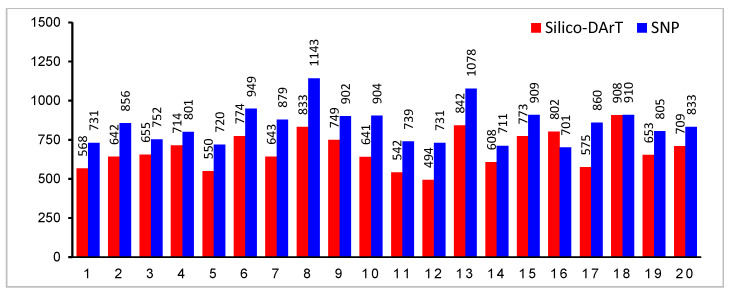
Distribution of filtered molecular markers across all soybean chromosomes. X-axis represents soybean chromosomes; Y-axis represents total number of markers across chromosomes.

DArTseq analysis yielded a total of 59,850 molecular markers including 18,540 Silico-DArT and 41,310 DArT-SNPs. After quality control, 13,765 Silico-DArT and 16,914 DArT-SNP markers remained for further research. The distribution of molecular markers across soybean chromosomes was illustrated in Figure 5, ranging nearly from 500 to 900 for Silico-DArT and from 700 to 1100 for SNPs. All remaining markers had an average read depth of 19, reproducibility of 0.995, and a call rate of 0.96

### 3.3. Population Structure Analysis

**Figure 6:**
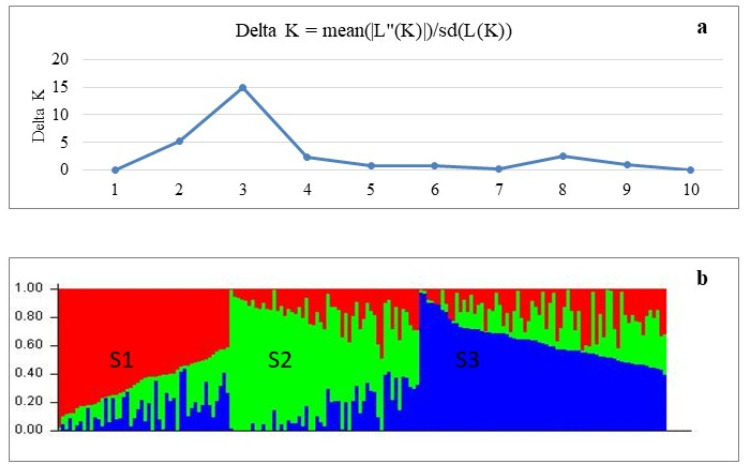
Population structure analysis of studied soybean accessions. (**a**) Delta K values for different numbers of populations assumed (K) in the STRUCTURE analysis. (**b**) Classification of soybean accessions into three sub-populations (K = 3) using STRUCTURE 2.3.3. The distribution of the accessions to different populations is indicated by the colour code. Numbers on the Y-axis show subgroup membership, whereas the X-axis shows the distribution of 170 studied soybean genotypes.

In population structure analysis, 170 soybean genotypes were divided into three groups based on the optimal delta K (ΔK) values (Figure 6). Subgroup S1 contained 48 genotypes belonging to maturity group MG 00-I, and 10 of them were considered admixed. The remaining genotypes in the subgroup S1 mainly originated from North America (5), Russia (15) and other European countries (15). The largest proportion of the genotypes in the subgroup S2 belong to MG II–III, and most of these originated from Kazakhstan (15) and Europe (21), among which 13 genotypes were admixed. Subgroup S3 consisted of 62 MG 00-I genotypes including 12 admixtures, and genotypes in the subgroup S3 were mainly from countries in Asia, North America and Europe.

### 3.4. GWAS Analysis, QTL and Candidate Gene Prediction

**Figure 7:**
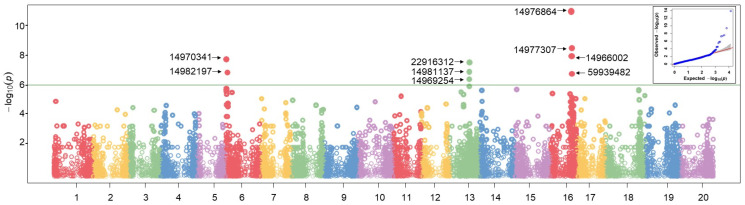
Manhattan plot of association mapping conducted using GLM. X-axis represents chromosome positions, Y-axis represents log10 (*p*-values). The green line in the Manhattan plot indicates the threshold (*p*-value < 1 × 10^−6^). The dots with numbers located above the threshold line represents QTLs that were significantly associated with PD. The Q-Q plot for the GLM model is located in the top-right corner.

GWAS was conducted utilising 170 soybean accessions evaluated across four distinct environments (Figure 7).

Five analytical models, including 2 single-locus and 3 multi-locus models, were used to find QTLs associated with PD and other pod-related traits. This multi-model strategy enabled a more reliable identification of marker-trait associations, elucidating the genetic background of pod dehiscence in soybean. GWAS results were illustrated using Manhattan plots, and Figure 7 shows an example of GWAS results where LOD score ≥ 6.0 used as a threshold.

According to the GWAS results, 48 QTLs were identified across 14 chromosomes (Supplementary Table S2). The majority of these identified QTLs were located on chromosome 16 (14 QTLs), followed by chromosomes 13 and 6, with 9 and 8 QTLs, respectively.

QTLs identified at least twice by different GWAS models, or detected in more than two environments, were considered as consistent and used for further investigation. As a result, 14 consistent QTLs were identified: eight QTLs for PD, two for PL, one for PW, one for WTR, one for RWCP and one for Ca+b. Subsequently, candidate genes were mined in the 20 Kb genomic flanking regions of identified QTLs. However, in most cases, candidate genes were selected if the identified SNP was located within the gene. As a result, 14 genes associated with the pod-related traits and physiological parameters were identified in studied soybean genotypes (Table 2).

Particularly, three stable QTLs were revealed on chromosome 16, corresponding to genes encoding a C2H2 zinc finger protein, methyl esterase 1, and a mitochondrial phosphate carrier protein, indicating a major role of this genetic region in PD resistance. Additionally, two tightly linked PD candidate genes, coding for Pollen Defective in Guidance 1 protein, isoform X1 (Glyma.13G184600) and DNA-binding bromodomain-containing protein (Glyma.13G184500), were identified on chromosome 13. There were also two genes related to PD on chromosome 6 including genes Glyma.06G011600 (Protein BPS1) and Glyma.06G034500 (RING finger protein 5). Two putative genes, Glyma.13G039900 (Receptor-like kinase) and Glyma.16G025800 (Nucleotidyltransferase), were found for PL on chromosomes 13 and 16, respectively. One QTL linked to hydroxyproline-rich glycoprotein gene was found on chromosome 6, while the pod-related trait WTR was associated with a major intrinsic protein family transporter on chromosome 19. RWCP was linked to Cycloartenol synthase 1 on chromosome 1. Finally, a candidate gene encoding a Kunitz trypsin inhibitor for Ca+b was identified on chromosome 19 (Table 2).

### 3.5. Genomic Prediction (GP) for Pod Dehiscence

GP for pod dehiscence was performed across two marker sets, the whole set of markers and GWAS-derived markers, and two types of GP strategy were employed: cross-prediction and across-prediction. Cross-prediction was conducted using cross validation methods, where the population was divided into a training set (80%) and testing set (20%). While in across-prediction, models were trained in one population and tested in a different population.

**Figure 8:**
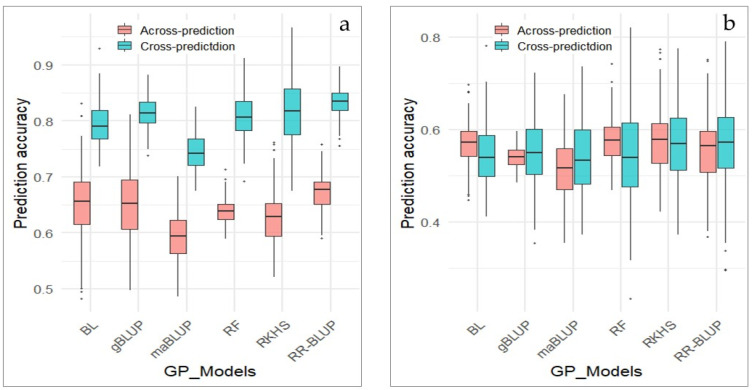
Genomic prediction of pod dehiscence using the whole and GWAS-derived marker sets. (**a**) GP of PD based on the whole markers; (**b**) GP of PD based on GWAS-derived markers. X-axis represents GP models, Y-axis represents prediction accuracy.

In this study, GP was employed using six models including BL, gBLUP, maBLUP, RF, RKHS and RR-BLUP. GP was yielded for prediction accuracy ranging from 0.74 to 0.83, when it was conducted with cross validation methods (cross-prediction) in the whole marker set (Figure 8a). With the exception of maBLUP, all five other models showed nearly the same prediction accuracy of slightly above 0.80. The accuracy of GP decreased over a range 0.59–0.67 compared to cross-prediction, when across-prediction was performed using the whole marker set.

The accuracy prediction ranged from 0.544 to 0.57 in cross-prediction and from 0.543 to 0.58 in across-prediction, when the GWAS-derived marker set was implemented in GP (Figure 8b). Overall, the prediction accuracy in both cross-prediction and across-prediction remained roughly unchanged. This finding suggests that all GP models based on both whole and GWAS-derived marker sets are effective for selection of favorable genotypes resistant to PD.

Finally, GP yielded genomic breeding values (GBV) for each genotype ranging from −45 to 19.0 units. This could help to select genotypes with best resistance and sensitivity to PD. Thus, based on GP, eight PD resistant genotypes were identified with low GBV: 186.1, Jevrika, Zen, Malvina, Zakat, VNIIS1, Sponsor and 209.1. In contrast, ten genotypes with highest GBV were selected as sensitive to PD, including: Dina, Fora, Warsawska, PJeP26, Carola, Nawiko, Kollekcyina, PJeP27, Svetlaia and Jaselda.

## 4. Discussion

Pod dehiscence is one of the main traits that can dramatically reduce soybean yield. In Kazakhstan, soybean is cultivated mainly in the south-east of the country where the summer climate is dry and hot, and this can increase the likelihood of PD during harvest. Therefore, it is crucial to develop non-dehiscent soybean cultivars adapted to the continental climate of South-Eastern Kazakhstan.

PD is caused by many factors, including morphological and physiological characters such as PL, PW RWC in pods, and other pod-related traits [12,80]. In this study, pod-related traits that have a significant effect on PD levels in soybean were investigated. The values of PL, PW and LWR in genotypes highly resistant to PD were found to be significantly lower than in soybean genotypes sensitive to PD (Figure 3). Similar results were reported earlier confirming that PL, PW, LWR and WTR were reliable indicators for assessing the PD level in soybean, and that the longer and thicker the pods were, the more sensitive they were to PD [13,81].

Further, significant positive correlation of PD with PL, LTR, WTR and RWCP was revealed in the current study (Figure 4). This result was supported by the recent publication in common bean where positive associations of PD with PL, PW, LTR and WTR were reported [14]. Furthermore, soybean genotypes sensitive to PD were reported to have longer PL and wider PW [21]. Additionally, the water content in pod cell walls plays an important role on PD because higher moisture content in pod cell walls has been observed in soybean genotypes resistant to PD [11,12,82].

In the current study, a negative correlation between PD and Cb was found which could potentially point to an important effect of photosynthetic pigments on PD. This kind of negative association was consistent with a recent report [30] and also supported by previous studies [12,83]. According to another published paper, soybean plants with a strong level of pod dehiscence had higher photosynthetic activity during seed development stages R5 and R6. However, the reduction of photosynthesis can act as a signal for the conversion of metabolites and build-up of cellulose, lignin and total fiber in pod cells [21]. Carbohydrates produced by photosynthesis are essential for cellulose synthesis, which is considered as one of the main enzymatic pathways important for the PD trait [84].

Positive correlations of PD with both SOD and CAT were observed in a recent study, which also reported that ROS levels can increase during PD [30]. Phases R5 and R6 represent the most vulnerable pod-setting stages in the development of soybean plants in response to drought and heat stresses [85,86]. Therefore, increased activity of antioxidant enzymes in leaves can help to support resistance of plants to unfavourable conditions. In mature plants in pod maturation stage R8, all metabolic processes were completed and the level of antioxidant enzymes decreased [87]. Our assessment of SOD and PPO antioxidant enzyme activity in soybean leaves in R5 and R6 stages did not reveal a direct relationship with PD. However, it might have indirect effects on yield components in studied genotypes like the number of pods, size of pods, seed weight, etc., which ultimately can affect the PD trait.

For DArT analysis, more than 30,000 high-quality DArT molecular markers with sufficient read depth were utilized to perform GWAS in 170 soybean genotypes. Soybean accessions were split into three distinct sub-populations, S1, S2 and S3, based on population structure analysis with these DArT markers. Two sub-populations, S1 and S3, contained early-maturing soybean genotypes originating from different countries worldwide, which might indicate for local gene flow as reported earlier [88,89]. On the other hand, sub-population S2 contained genotypes from Kazakhstan and Europe with a middle-late maturity time, suggesting a more uniform background created by local breeding programs [90].

In the current study, GWAS conducted in 170 soybean genotypes revealed 48 QTLs, of which 14 were stable QTLs across different environments (Supplementary Table S2 and Table 2). Candidate genes linked to PD were located on chromosomes 6, 7, 13 and 16. Previously, the main QTL for PD had been detected on chromosome 16, namely *pdh1* (Glyma.16g141400) and *SHAT1-5* (Glyma16g019400) [9,36]. More recently, Li et al. [47] detected a new gene, *Sh1*, on chromosome 16, whereas another potential PD gene, Glyma.16g076600, was reported controlling the synthesis of a specific protein related to ABA catabolism [46]. In our study, three PD tentative candidate genes, Glyma.16G141100, Glyma.16G145100 and Glyma.16G146900, were found on chromosome 16. Among them, Glyma.16G141100 encoding the C2-H2 zinc finger protein was identified across all environments, and it was identical to the described *Sh1*. This *Sh1* gene (Glyma.16G141100) was located very close to *Pdh1* (Glyma.16G141400) and it was shown to down-regulate expression of *SHAT1-5* binding its promotor region, thickening secondary cell walls in lignified fiber cap cells [47].

Transcription factors (TF), bHLH, WRKY, NAC, MYB and MYB-related families, were shown to have a significant effect on regulating PD [91,92,93]. For instance, members of MYB families in *Arabidopsis* activate other TFs which can lead to PD by regulating secondary cell wall formation [94]. On chromosome 13, three DArT markers corresponding two genes (Glyma.13G184500 and Glyma.13G184600) were identified (Figure 7 and Table 2). Glyma.13G184500 encodes a MYB TF indicating that it might be a potential regulator in PD. However, this requires further functional validation to confirm its role in PD development. On chromosome 6, two stable DArT markers (Figure 7 and Table 2) and two SNPs (Supplementary Table S2) were identified. QTL_PD2 with Glyma.06G011600 was highly expressed in pods according to Libault et al. [95], while one of the SNPs in gene Glyma.06G012500 encoding 3-ketoacyl-CoA synthase 4 was tightly linked to Glyma.06G012200 from the bHLH family protein, which is an important TF for PD [92,96]. Other stable QTLs did not show any homology to known genes or TFs associated with PD. Nevertheless, two QTLs with tentative genes Glyma.03G033300 and Glyma.06G199800 were identified in only one environment. They were closely related to TFs for PD. For example, Glyma.03G033300 linked to PW was located next to the Glyma.03G033600 (C2H2 family protein), which is a crucial TF regulating PD in legumes including soybean [47,97]. Glyma.06G199800, corresponding to WTR, is in close proximity to Glyma.06G199600, representing a bHLH family protein. Such bHLH TF genes in *Arabidopsis* induce a non-lignified cell layer in the dehiscence zone, which leads to silique expansion [96,98].

Various GP models have been successfully used in evaluating complex agronomic traits and disease resistance. Genomic prediction is a very powerful approach that can dramatically accelerate the breeding process. Prediction accuracy in GP is important and it can be obtained from correlation between observed phenotypes and predicted genomic estimated breeding values (GEBVs) [99,100]. Prediction accuracy depends on model types as well as optimal marker set, which can be implemented in different random marker sets, ranging from several to thousands of markers and GWAS-derived best SNPs. For instance, salt tolerance in soybean germplasm was utilized using GP with various marker sets ranging from 10 to 10,000 [101]. In the result, the marker set with 10,000 SNPs was reported to show high prediction accuracy along with the 10 best GWAS-derived SNPs. In a similar investigation for GP of arginine content in soybean, it was concluded that marker sets with more SNPs and top SNPs derived from GWAS lead to better prediction accuracy in GP [102].

Apart from the optimal marker set, the training and testing populations also play an important role in prediction accuracy in GP. In general, models trained and tested within the same population produce higher accuracy compared to models trained and tested across different populations [102,103,104].

In the current study, two different marker sets were employed through cross-prediction and across prediction approaches. Genomic prediction models using different marker sets conducted in 170 soybean genotypes showed high prediction accuracies. Cross-prediction based on the whole set of markers resulted in higher prediction accuracy compared to GWAS-derived markers. However, when across-prediction was utilized on the whole marker set, prediction accuracy decreased from an average 0.80 to around 0.65. In contrast, GWAS-derived markers yielded similar prediction accuracy values (approximately 0.55) in both cross-prediction and across-prediction of PD. Across-prediction based on training models in one population and testing them in a new, unknown population, indicates that GWAS-derived marker set is more stable for selecting PD when evaluating new soybean genotypes. In addition, GP provide results for the selection of soybean genotypes most resistant as well as most sensitive to PD.

In general terms, for GP accuracy, there is no a ‘Golden standard’, as it varies widely depending on several factors, including the studied traits, population structure, marker density, and statistical models used. For example, in soybean, GP accuracy for the same trait can differ considerably across studies. For soybean seed weight, GP accuracies ranged between 0.75 and 0.87 [105]. It also was reported that average prediction accuracy of 0.39 for soybean yield [99], and up to 0.80 in some populations [106], for plant height 0.86 and yield per plant 0.47 [107], and for grain yield (0.58–0.60), plant height (0.43–0.45), and days to maturity (0.67–0.68) [106]. In the current study, the genomic prediction accuracy was approximately 0.60, indicating for good predictive performance and suggests that the model captured a substantial proportion of the genetic variance for the evaluated traits.

## 5. Conclusions

In this study, GWAS was carried out using single and multi-locus models to reveal QTLs corresponding to PD in soybean. As a result, 48 QTLs in total were identified with 14 stable QTLs, mostly located on chromosomes 6, 13 and 16, corresponding to pod dehiscence and pod-related traits. From putative candidate genes, two most stable and important genes for PD with known functions were emphasised from the QTLs: Glyma.13G184500 and Glyma.16G141100, encoding transcription factors, DNA-binding bromodomain-containing protein and C2H2 zinc finger protein, respectively. Additionally, genomic prediction (GP) was performed employing different marker sets. Cross-validation and across-prediction were used to determine the prediction accuracies of models. This demonstrated stability of GWAS-derived markers through across-prediction. Finally, GP helped to select the most PD resistant and sensitive soybean genotypes according to predicted genomic estimated breeding values. Therefore, the identified putative candidate genes will be used to improve the efficiency of marker-assisted selection for developing PD resistant varieties in soybean. Furthermore, assessed genomic prediction (GP) models for across-population prediction will be implemented to identify new PD resistant cultivars in future soybean breeding programs.

## Figures and Tables

**Table 1:** Mean, range and standard deviation for observed PD-related traits of 170 soybean genotypes grown in 2023–2024. PD, pod dehiscence; RWCP, Relative water content in pods; PL, pod length; PW, pod width; PT, pod thickness; LWR, length/width ratio; LTR, length/thickness ratio; WTR, width/thickness ratio; Ca, chlorophyll a; Cb, chlorophyll b; Cx+c, total carotenoids; SOD, superoxide dismutase; PPO, polyphenol oxidase.

Traits	Mean	Min ^1^	Max	SD	Mean Square
PD (%)	21.6	0	88.25	20.81	897.04 ***
RWCP (%)	92.23	85.47	95.9	1.69	8510.03 ***
PL (cm)	4.53	3.37	6.8	0.35	20.62 **
PW (cm)	0.93	0.7	1.21	0.06	0.88 *
PT (cm)	0.7	0.52	0.84	0.05	0.49 **
LWR	4.87	4.15	7.1	0.33	23.84 ***
LTR	6.54	5.17	10.11	0.62	43.16 ***
WTR	1.35	1.12	1.72	0.1	1.82 ***
Ca (mg/g)	1.88	1.34	2.85	0.27	3.61 **
Cb (mg/g)	0.63	0.41	1.12	0.12	0.41 **
Cx+c (mg/g)	0.51	0.21	0.69	0.1	0.27 ***
SOD (units/g FW)	98.63	43.5	141.4	19.44	10,103.16 ***
PPO (units/g FW)	66.83	11.25	296.88	48.59	6813.8 ***

^1^ Min, minimal value; Max, maximal value; SD, standard deviation; * *p* < 0.05; ** *p* < 0.01; *** *p* < 0.001.

**Table 2:** The 14 putative candidate genes identified by the stable markers associated with the studied pod dehiscence and related traits.

QTL	DArT Marker	Chr	Relevant Gene	Gene Position	Annotated Description
**Pod dehiscence, PD**					
QTL_PD2	14982197	6	Glyma.06G011600	866275-867210	DUF241 domain protein; protein BPS1
QTL_PD3	14970341	6	Glyma.06G034500	2677248-2679092	RING finger protein 5
QTL_PD6	22919889	7	Glyma.07G125300	14920233-14979323	DNA-directed RNA polymerase family protein
QTL_PD8	14969254	13	Glyma.13G184600	29837154-29848121	Pollen Defective in Guidance 1 protein, isoform X1
QTL-PD11	14981137	13	Glyma.13G184500	29829471-29834027	DNA-binding bromodomain- containing protein
QTL_PD14	50681234	16	Glyma.16G141100	29893194-29896920	C2H2 zinc finger protein
QTL_PD15	14976864	16	Glyma.16G145100	30578388-30581421	Methyl esterase 1
QTL_PD16	14976250	16	Glyma.16G146900	30763467-30766039	Mitochondrial phosphate carrier protein 3
**Pod length, PL**					
QTL1_PL3	22917169	13	Glyma.13G039900	12319075-12321626	Receptor-like kinase 1
QTL2_PL4	22919231	16	Glyma.16G025800	2499822-2508300	Nucleotidyltransferase
**Pod width, PW**					
QTL_PW2	100064442	6	Glyma.06G013500	1009335-1010213	Hydroxyproline-rich glycoprotein family protein
**Width/thickness ratio of pods, WTR**					
QTL-WTR2	50680953	19	Glyma.19G123600	38159905-38162342	Major intrinsic protein (MIP) family transporter
**Relative water content in pods, RWCP**					
QTL_RWCP1	14970004	1	Glyma.01G001300	227021-237987	Cycloartenol synthase 1
**Chlorophyll a and b, Ca+b**					
QTL1_Ca+b	22914385	9	Glyma.09G163700	38808617-38809561	Kunitz trypsin inhibitor 1

## Data Availability

The data presented in the manuscript are available in the Supplementary Materials.

## Supplementary Materials

The following supporting information can be downloaded at: https://www.mdpi.com/article/10.3390/plants14223505/s1, Table S1: Information about used soybean accessions: Names, maturity groups and origin; Table S2: Identified QTLs and putative genes associated with studied traits.
